# Impact of time-varying cumulative bevacizumab exposures on survival: re-analysis of data from randomized clinical trial in patients with metastatic colo-rectal cancer

**DOI:** 10.1186/s12874-020-01202-9

**Published:** 2021-01-09

**Authors:** Adrien Guilloteau, Michal Abrahamowicz, Olayide Boussari, Valérie Jooste, Thomas Aparicio, Catherine Quantin, Karine Le Malicot, Christine Binquet

**Affiliations:** 1 INSERM, U1231, EPICAD team, Equipe EPICAD, 7, Bld Jeanne d’Arc, Dijon, France; 2 University of Bourgogne Franche-Comté, UMR “Lipides Nutrition Cancer”, EPICAD team, Dijon, France; 3 Infection Control Unit, Dijon-Bourgogne University Hospital, Dijon, France; 4 Department of Epidemiology, Biostatistics and Occupational Health, McGill University, Montreal, QC Canada; 5 Digestive Cancer Registry of Burgundy, Dijon-Bourgogne University Hospital, Dijon, France; 6 Fédération française de cancérologie digestive (FFCD), Biostatistics department, Dijon, France; 7 Assistance Publique - Hôpitaux de Paris, Saint-Louis Hospital, Gastroenterology and Digestive Oncology Department, Université Paris 7, Sorbonne Paris Cité, Paris, France; 8 Biostatistics and Bioinformatics (DIM), Dijon-Bourgogne University Hospital, Dijon, France; 9 Inserm, CIC1432, Clinical Epidemiology unit, Dijon, France; 10 Dijon-Bourgogne University Hospital, Centre d’investigation clinique, Module Epidémiologie Clinique/Essais cliniques Dijon, Dijon, France; 11 INSERM, U1181, Paris France ; Université Paris-Saclay, B2PHI Paris, France

**Keywords:** Time varying cumulative exposure to maintenance treatment, Survival, Colorectal cancer, Bevacizumab

## Abstract

**Background:**

As cancer treatment, biotherapies can be as effective as chemotherapy while reducing the risk of secondary effects, so that they can be taken over longer periods than conventional chemotherapy. Thus, some trials aimed at assessing the benefit of maintaining biotherapies during chemotherapy-free intervals (CFI). For example, the recent PRODIGE9 trial assessed the effect of maintaining bevacizumab during CFI in metastatic colorectal cancer (mCRC) patients. However, its analysis was hindered by a small difference of exposure to the treatment between the randomized groups and by a large proportion of early drop outs, leading to a potentially unbalanced distribution of confounding factors among the trial completers. To address these limitations, we re-analyzed the PRODIGE9 data to assess the effects of different exposure metrics on all-cause mortality of patients with mCRC using methods originally developed for observational studies.

**Methods:**

To account for the actual patterns of drug use by individual patients and for possible cumulative effects, we used five alternative time-varying exposure metrics: (i) cumulative dose, (ii) quantiles of the cumulative dose, (iii) standardized cumulative dose, (iv) Theoretical Blood Concentration (TBC), and (v) Weighted Cumulative Exposure (WCE). The last two metrics account for the timing of drug use. Treatment effects were estimated using adjusted Hazard Ratio from multivariable Cox proportional hazards models.

**Results:**

After excluding 112 patients who died during the induction period, we analyzed data on 382 patients, among whom 320 (83.8%) died. All time-varying exposures improved substantially the model’s fit to data, relative to using only the time-invariant randomization group. All exposures indicated a protective effect for higher cumulative bevacizumab doses. The best-fitting WCE and TBC models accounted for both the cumulative effects and the different impact of doses taken at different times.

**Conclusions:**

All time-varying analyses, regardless of the exposure metric used, consistently suggested protective effects of higher cumulative bevacizumab doses. However, the results may partly reflect the presence of a confusion bias. Complementing the main ITT analysis of maintenance trials with an analysis of potential cumulative effects of treatment actually taken can provide new insights, but the results must be interpreted with caution because they do not benefit from the randomization.

**Trial registration:**

clinicaltrials.gov, NCT00952029. Registered 8 August 2009.

**Supplementary Information:**

The online version contains supplementary material available at 10.1186/s12874-020-01202-9.

## Background

Randomized Control Trials (RCT) are the gold-standard design to assess the causal effect of any intervention in part due to the comparability of randomized groups. Recently, RCT’s of cancer treatments have evolved to better take into account the specificities of targeted treatment of cancers, like monoclonal antibodies targeting pathways that are crucial for tumour growth cells tumor. As these therapies have significantly fewer side effects than classical chemotherapy, they can be administered for longer time periods and without treatment interruptions [1–3]. For these reasons, recent “maintenance trials” have aimed at evaluating the efficacy of a continuous administration of immunotherapies in advanced cancers, compared to their use only during the chemotherapy sequence [1, 4, 5]. In such trials, all participants receive the same chemotherapy and the difference between the interventions received by different trial arms is limited to whether the additional targeted treatment is received or not during the Chemotherapy Free Interval(s) (CFI) [1, 6].

However, similar to RCT in other fields, maintenance trials, might be subject to increasing bias as their length of follow-up increases [7]. Indeed, results of standard Intention-to-Treat (ITT) analyses may be affected by drops out and/or decreasing adherence to protocol and changes in dose or treatments, possibly related to side effects or patient’s overall condition, all of which become more likely with increasing follow-up time [7–11]. The resulting changes over time in the actual use of the drug at study imply that the conventional ITT analyses reliance on both: (a) time-invariant exposure, defined by randomization groups, and (b) assumption of a constant-over-time treatment effect, underlying the very popular proportional hazards model [12], is questionable. Therefore, we hypothesize that, in maintenance trials, the use of methods developed for observational studies of drug effectiveness or safety, that account for time-varying nature of treatment exposure and/or its possibly cumulative effects, may help avoid the aforementioned limitations of ITT analyses and provide insights regarding the way a specific biotherapy may possibly improve patients’ prognosis or not. In addition, in cancer trials, accounting for cumulative exposure may be especially appropriate, because of the complexity of mechanisms involved in the treatment effect and the fact that the drug can accumulate in the tissue [13, 14]. In the past decade, several methods have been developed to allow modeling cumulative effects of time-varying exposures [15–23]. However, these methods have rarely been applied in post-hoc analyses of RCT and, to the best of our knowledge, no study investigated the advantages and limitations of different time-varying exposure metrics in this context.

In this study, we aimed at an empirical real-life comparison of alternative ways to model effects of a time-varying treatment, in terms of the model’s fit to data, the estimated treatment effect, and the resulting conclusions. To this end, we re-analyzed the recent results of the PRODIGE 9 trial, a maintenance randomized clinical trial of the potential effect of bevacizumab, a monoclonal antibody targeting the V*ascular Endothelial Growth Factor* (VEGF), on delaying all-cause mortality in metastatic colorectal cancer [6].

## Methods

### Data source: PRODIGE 9 trial

PRODIGE 9 is an open label randomized phase 3 maintenance trial [6, 24]. The trial randomized 494 patients newly diagnosed with a histologically proven, unresectable metastatic colorectal cancer (mCRC), between March 2010 and July 2013, in one of the 66 participating centers in France. Inclusion criteria included life expectancy greater than 3 months, and no previous chemotherapy or anti-angiogenic therapy for metastatic disease. Consenting participants were first stratified according to: study site, previous primary tumor resection and Köhne prognostic classification (good, intermediate or poor), and then assigned, within each stratum, to either the maintenance or the CFI arm, using simple 1:1 randomization [6].

The original aim was to compare (a) bevacizumab during CFI versus (b) no treatment during CFI after an induction sequence with FOLFIRI (folinic acid, 5-fluoro-Uracile and irinotecan) combined with bevacizumab (5 mg/kg every 2 weeks). The main outcome was tumor control duration, defined as the time to tumor progression (diagnosed on CT-scan according to the Response Evaluation Criteria in Solid Tumors) during a sequence of chemotherapy [6]. Patients who died without progression were censored.

The induction sequence lasted 12 treatment cycles (24 weeks), followed by a CFI whose length was determined by the clinical state of individual patients [6]. For both groups, a new sequence of chemotherapy of 16 weeks (8 treatment cycles) began after the CFI in case of progression or investigator-based decision. Patients underwent CFI and chemotherapy alternatively until they left the study protocol [6].

Outcomes that occurred until December 2016 were include in the study. Sociodemographic characteristics, tumor characteristics (localization, size, primitive tumor resection, KRAS, NRAS and BRAF status) were assessed at randomization. A standard examination (including WHO Performance Status (PS) and biological samples) associated with a CT scan to assess signs of progression according to RECIST criteria [25], and toxicity evaluation were repeated every 8 weeks during the treatment protocol [6]. The reported ITT analyses yielded generally negative results with no evidence of systematic differences in median tumor control duration (HR=1.07 for maintenance with the control arm as reference; 95%-CI=0.85–1.34; *p*=0.57], progression free survival (HR=0.91; 95%-CI=0.76–1.09; *p*=0.316) or overall survival (HR=1.07; 95%-CI=0.88–1.29; *p*=0.500) [6]. The original PRODIGE 9 trial was approved by the Committee for the Protection of Persons Ile de France VIII and was registered on clinicaltrials.gov (NCT00952029) [6].

### Statistical analysis

Our re- analyses of PRODIGE9 data relied on statistical methods for survival (time-to-event) analyses, and used death of any cause as the endpoint. Because the PRODIGE 9 protocol did not differ between the two groups during the initial 6-month induction sequence [6], we shifted time 0 (baseline) to 6 months after randomization, the expected date of the beginning of the first CFI. Accordingly, our analyses were limited to only those patients who remained alive until 6 months post-randomization. The main objectives of the re-analyses was to explore potential benefits of using time-varying exposure metrics and accounting for possibly cumulative effects of bevacizumab treatment. To this end, we compared how the estimated associations with the overall survival varied across five alternative time-varying bevacizumab exposure metrics, included in multivariable Cox proportional hazards (PH) model and its flexible extensions [22], using either the exposure to bevacizumab administered only during CFI (CFI exposure) or exposure during all the study protocol including in the induction sequence (overall exposure). We then contrasted their results with the conventional ITT analysis that defined a time-invariant exposure as binary indicator of randomization group (model 1).

Models 2a (for CFI exposure) and 2b (for overall exposure) defined the time-varying metric as a continuous variable (CE) representing the updated current value of the cumulative administrated dose at any time t, calculated as the sum of all doses received until a given time *t*:

1 $$ {CE}_i(t)={\sum}_{t_0<\dots <{t}_k<\dots <t}^t{X}_i\left({t}_k\right) $$

with *X*_*i*_(*t*_*k*_), the dose received at time t_k_ by patient i; t_0,_ the time of origin;

Models 3a (for CFI exposure) and 3b (for overall exposure) relied on a categorical variable (CEQ), defined by quantiles of the distribution of the cumulative dose *CE*_*i*_(*t*) in [1].

2 $$ {\mathrm{C}\mathrm{EQ}}_{\mathrm{i}}\left(\mathrm{t}\right)={\sum}_{\mathrm{k}=1}^{\mathrm{p}}\mathrm{k}\times {\mathrm{I}}_{\left\{\mathrm{E}{\mathrm{C}}_{\mathrm{i}}\left(\mathrm{t}\right)\in {\mathrm{A}}_{\mathrm{k}}\right\}} $$

with A_k_ corresponding to the k^eme^ quartile range for *p*=4 (k^eme^ tertile range for *p*=3, respectively).

Model 3a consisted in five categories corresponding to (i) the control group (reference category) (ii) patients of the maintenance group who did not receive any bevacizumab at time t (iii)-(v) tertiles of the updated cumulative dose CE(t) among those subjects who had non-zero cumulative dose at a given time. Model 3b consisted in four categories corresponding to the quartiles of the updated cumulative dose CE(t) among those subjects who had non-zero cumulative dose at a given time.

Models 4a (for CFI exposure) and 4b (for overall exposure) used the updated standardized cumulative dose (StCE), obtained by converting the values of the cumulative dose CE(t) in [1] observed for individual subjects at time t into z-scores:

3 $$ {\mathrm{StCE}}_{\mathrm{i}}\left(\mathrm{t}\right)=\frac{{\mathrm{EC}}_{\mathrm{i}}\left(\mathrm{t}\right)-\overline{\mathrm{EC}\left(\mathrm{t}\right)\ }}{\upsigma_{\mathrm{EC}\left(\mathrm{t}\right)}} $$

where $\overline{EC(t)\ }$ is the mean of the cumulative doses at time *t* and *σ*_*EC*(*t*)_ is their standard deviation*.*

This approach eliminated the systematic differences between the values of cumulative doses CE(t) in [1], calculated at different times during the follow-up.

Model 5 defined the time-varying exposure metric as the expected theoretical blood concentration (TBC) of bevacizumab, at time *t*, for a given individual. This metrics is defined as the weighted sum of past bevacizumab doses received by the patient, with weights following the exponential decay model [26]. The weights were calculated assuming the half-life of bevacizumab was h = 20 days, based on previous pharmaco-kinetics studies [27].

4 $$ {TBC}_i(t)={\sum}_{t0<\dots <{t}_k<\dots <t}^t{X}_i\left({t}_k\right)\times 0,{5}^{\left(\frac{t_k-t}{h}\right)} $$

Finally, model 6 relied on a weighted cumulative exposure (WCE) metric, in which weights also depend on the time elapsed since the dose was taken *(t*_*k*_ *– t)* [15]. However, in contrast to model 5 in [4], in the WCE model the weights are estimated directly from the data, using a very flexible cubic spline model, that requires only minimal assumptions, resulting in a weight function w *(t*_*k*_ *– t)* that is continuous and smooth, but can take an arbitrary shape [22]:

5 $$ {WCE}_i(t)={\sum}_{t0<\dots <{t}_k<\dots <t}^t{X}_i\left({t}_k\right)\times w\left({t}_k-t\right) $$

The use of un-penalized cubic regression splines is based on several earlier statistical papers that have built the WCE methodology and validated it in comprehensive simulations [22, 16, 28, 29]. This approach combines sufficient flexibility to recover a wide range of functional shapes (as demonstrated in simulations) with ease of statistical inference (due to use of un-penalized maximum likelihood estimation) and is implemented in the R program [30]. The underlying assumptions are that (i) the weight function is a smooth function (with continuous 1st and 2nd derivatives) of time elapsed between the exposure and the current time when the risk is being assessed; and (ii) this function can take an arbitrary shape, including both monotone and non-monotone curves, and (iii) can take positive (indicating risk increases) and/or negative (risk decrease) values for different times in the past [22]. Because of uncertainty regarding the maximum duration of the effect of past exposures, we estimated three alternative WCE models with exposure windows of 120, 365 (1 year) or 730 (2 years) days, respectively, and selected the best-fitting WCE model based on the minimum AIC [22]. Adjusted Hazard Ratios corresponding to some clinically plausible bevacizumab exposure patterns were reconstructed based on the estimated weight function, as described by Sylvestre & Abrahamowicz 2009 [22].

Section 1 of Online Appendix describes how we tested for the linearity and proportional hazards (PH) hypotheses. Goodness of fit of alternative models was compared through the Akaike Information Criterion (AIC), a decrease of 4 points was deemed a moderate improvement, and a decrease of more than 10 points was deemed important [31].

Because time-varying changes in bevacizumab use during the follow-up were based on multiple factors (patient general condition, tumor evolution, patient and investigator choices …), we had to control for potential confounding bias. To ensure the comparability of results, all multivariable models adjusted the bevacizumab exposure for the same a priori selected potential confounders. Adjustment variables included both time-invariant covariates (age at randomization, sex, group of randomization, resection of the primitive tumor, initial level of phosphatase alkaline, localization of the primitive tumor), and time-varying variables (updated values of WHO performance status, a binary indicator of any toxicities related to bevacizumab, as well as updated values of weight, hemoglobin concentration, bilirubin concentration, blood pressure level). A directed acyclic graph representing the main risk factors and disease history in patients with colorectal metastatic cancer in the PRODIGE 9 study is presented in Appendix 2.

In sensitivity analyses, we assessed the effect of adjusting for time-varying variables. To this end, we re-estimated all the models adjusting only on the baseline values of all aforementioned time-varying variables. In further sensitivity analyses, in the TBC and WCE models we adjusted for an additional time-dependent binary indicator of “having received a treatment dose in the last 20 days”. The goal was to reduce concerns about the potential reverse causality bias that could occur if having received any dose recently may be a marker very poor health indicating a patient is likely to die very soon.

## Results

### Study population

A total of 106 patients (22.7%) were excluded because they died (38 patients) or experienced progression before 6 months (54 patients) after randomization, were lost to follow-up (one patient), or did not have complete information for relevant covariates (13 patients). Thus, our re-analyses were limited to 382 (77.3%) of the 488 patients included in the original ITT analyses [6]. Appendix 3 shows the flow chart for patients’ selection with a table presenting characteristics of excluded and included patients (Appendix 4). Patient characteristics are compared between the two trial arms, in Table 1.

**Table 1:** Baseline characteristics of 382 patients included in the re-analyses of the PRODIGE 9 Trial

	Maintenance group	Control group
**Sample size**	193	189
**Patients characteristics**		
Age		
< 65 years old	102 (52.8%)	97 (51.3%)
≥ 65 & < 75 years old	59 (30.6%)	58 (30.7%)
≥ 75 years old	32 (16.6%)	34 (18.0%)
Women	71 (36.8%)	62 (32.8%)
WHO performance status (PS)		
0	105 (54.4%)	102 (54.0%)
1	81 (42.0%)	78 (41.3%)
2	7 (3.6%)	9 (4.8%)
Alkaline phosphatase > 300 U/L	33 (17.1%)	35 (18.5%)
Leucocytes > 10 × 10^9^	51 (26.4%)	45 (23.8%)
Localisation		
Left colon	65 (33.7%)	60 (31.7%)
Right and transverse colon	38 (19.7%)	49 (25.9%)
Unspecified colon	42 (21.8%)	47 (24.9%)
Rectum	48 (24.9%)	33 (17.5%)

Among the 382 patients, 320 (83.8%) died during the follow-up (83.9% in the maintenance group vs 83.6% in the control group). For those who died, the median time from the baseline (shifted to 6 months after randomization) to death was 16.4 months (IQR: 8.9–26.7). The median follow-up of patients who remained alive until the end of the follow-up was 42.2 months (IQR: 34.4–48.6). Almost half of the 382 patients had their treatment stopped (bevacizumab and FOLFIRI chemotherapy) for other reasons than progression or death (28.7% by decision of the investigator, 8.3% due to toxicities, 11.3% due to other non-clinical reasons. These patients’ outcomes contributed to the analysis until their times of death or the end of the follow-up.

### Distributions of cumulative and standardized exposures

**Fig. 1:**
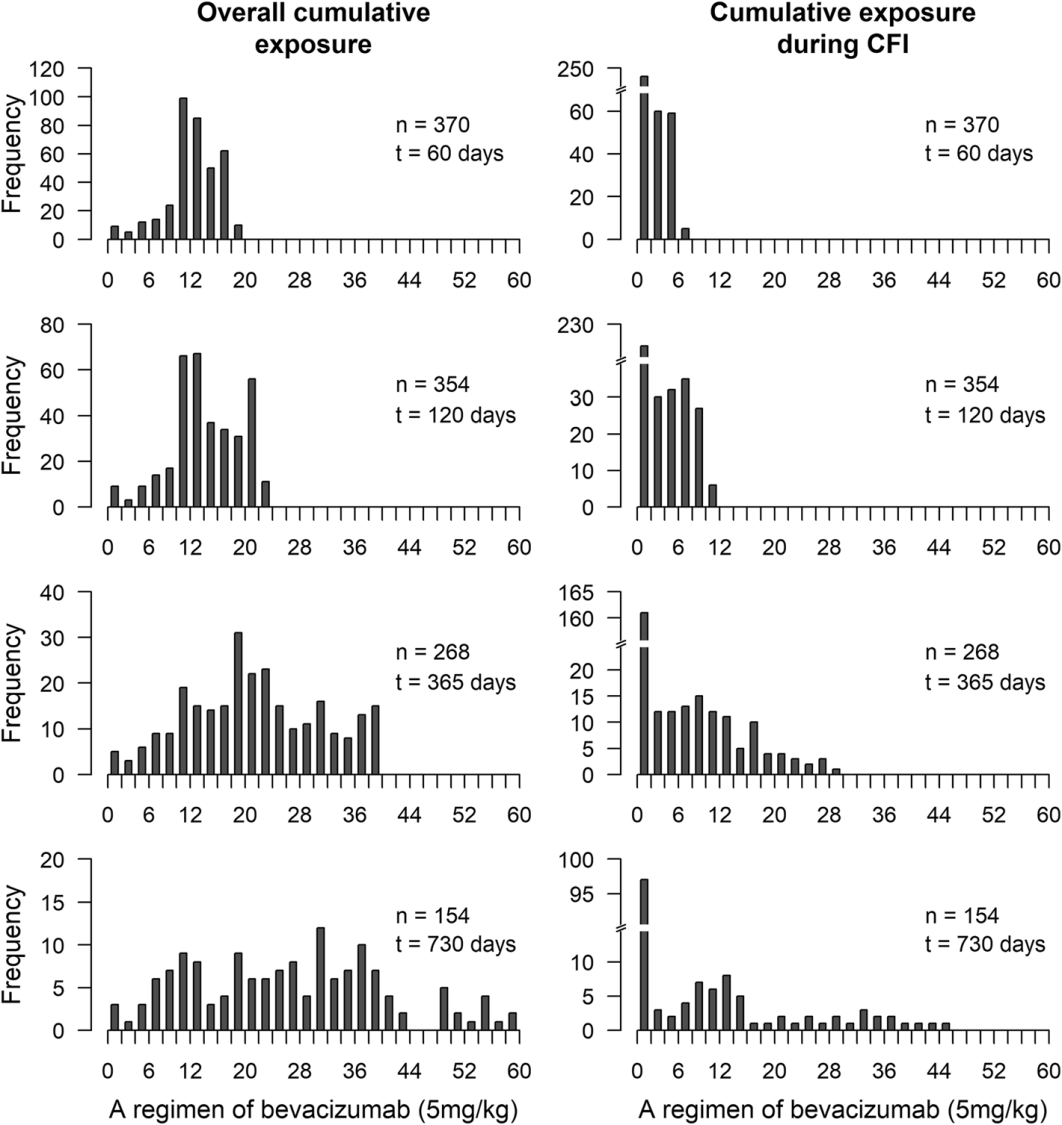
Cumulative exposure to bevacizumab at 60, 120, 182, 365 and 730 days after baseline. One unit on the x-axis is equivalent to the dose of bevacizumab received during one cure (5 mg/kg)

The distributions of the updated values of cumulative (CE) and standardized (StCE) exposures to bevacizumab at different time points are, respectively, presented in Fig. 1 and in Appendix 5.

Figure 1 shows that, as expected, the variance of the updated values of the cumulative bevacizumab dose increased considerably with increasing follow-up duration. In contrast, Appendix 5 shows that the distribution of the standardized cumulative dose remains approximately stable across the follow-up.

### Association between cumulative doses and overall survival

Table 2 summarizes the results of ITT analyses (model 1) and alternative time-varying models that account for un-weighted cumulative bevacizumab. Doses respectively, during CFI (upper half of the table) and for the overall exposures (lower half).

**Table 2:** Results of the intention to treat analysis and models time-varying cumulative exposure to bevacizumab during Chemotherapy Free-Interval (CFI) and overall cumulative exposure to bevacizumab (Prodige 9 reanalysis; *n*= 382)

	ITT analysis Traitement group (TG)	TG + time-varying cumulative dose [1]	TG+ categorized time-varying cumulative dose [2]	TG + time-varying standardize cumulative dose [3]
**Cumulative exposure during CFI**	**Model 1** HR [95% CI)	**Model 2a** HR [95%-CI]	**Model 3a** HR [95%-CIl]	**Model 4a** HR [95%-CIl]
Control (ref.)	1	1	1	1
Maintenance group	1.14 [0.90–1.44]	**1.45 [1.09–1.91]**	–	**1.68 [1.25–2.24]**
Cumulative dose [1]		**0.98 [0.96–0.99]**		
Maintenance - No dose received			0.94 [0.61–1.44]	
Maintenance - T1			**1.79 [1.32–2.44]**	
Maintenance - T2			1.23 [0.87–1.75]	
Maintenance - T3			**0.50 [0.30–0.83]**	
Standardized dose [3]				**0.69 [0.58–0.83]**
*AIC*	*2208.2*	*2200.4*	*2187.2*	*2192.4*
*Global Grambsch test*	*0.75*	***0.03***	*0.14*	*0.24*
**Overall cumulative exposure**		**Model 2b** HR [95%-confidence interval]	**Model 3b** HR [95%-confidence interval]	**Model 4b** HR [95%-confidence interval]
Control (ref.)		1	1	1
Maintenance group		1.27 [0.98–1.63]	**1.56 [1.20–2.02]**	**1.37 [1.07–1.77]**
Cumulative dose [1]		**0.99 [0.98–1.00]**		
Maintenance - Q1			1	
Maintenance - Q2			**1.58 [1.15–2.16]**	
Maintenance - Q3			0.92 [0.63–1.35]	
Maintenance - Q4			**0.50 [0.32–0.78]**	
Standardized dose [3]				**0.76 [0.66–0.88]**
*AIC*		*2205.7*	*2180.5*	*2196.7*
*Global Grambsch test*		*0.12*	*0.09*	*0.28*

*CI* Confidence Interval, *AIC* Akaike Information Criterion, *CFI* Chemotherapy Free Interval, *T1/2/3* Tertiles of the time-varying distribution of cumulative doses of bevacizumab, *Q1/2/3/4* Quartile of the time-varying distribution of cumulative doses of bevacizumab

Model 1 (leftmost column) suggests a slight, statistically non-significant increase in the all-cause mortality in the maintenance group. However, these results are based on conventional ITT-like analyses, that relies on time-invariant exposure, defined by the initial randomization and, thus, ignore between-patient differences in the actual exposure. On the other hand, all other models in Table 2 account for the time-varying exposures and estimate associations with different cumulative exposure metrics. Because models 2 and 4 adjust for cumulative bevacizumab doses, the corresponding Hazard Ratios [HR] for the maintenance group compare the mortality hazards (i) specific to those patients in this group who never got any bevacizumab doses until a given time vs. (ii) patients in the control group. The resulting adjusted HR’s are considerably higher than the HR for the maintenance group in Model 1, suggesting that those patients who never got bevacizumab in spite of being randomized to maintenance treatment are at especially high risk of death. In contrast, in time-varying models 2 and 4, among patients in the maintenance group, higher cumulative bevacizumab doses are associated with mortality hazard (HR< 1), with 95%-Confidence Intervals [CI] that often exclude 1.0 (Table 2). Together, these results demonstrate that the HR in the conventional Model 1, common to all patients in the maintenance group, represents a mixture of two different effects (a) risk increase for those who did not receive bevacizumab doses, and (b) gradually decreasing risks associated with higher cumulative bevacizumab dose. Similar picture emerges from the results of Models 3a and 3b that relied on tertiles of the cumulative dose. For example, model 3a indicates that patients in the maintenance group in the highest tertile of the cumulative dose had 50% *lower* hazard than those in the control group (HR=0.50, 95% CI: 0.32–0.78).

#### Theoretical blood concentration (TBC)

According to the TBC exponential decay model with a half-life h=20 days ([4]), a patient with a good adherence to the study protocol will have between 8 and 13 mg/kg of cumulative bevacizumab theorical blood concentration after 3 consecutive doses received 15 days apart (see Figure A and Table 1 in Appendix 7). Models using TBC of bevacizumab yielded the best-fit among all the models considered (Table 3, models 5–6), with AIC decreasing by at least 80 points relative to all simpler unweighted models in Table 2.

**Table 3:** results of the Theorical Blood Concentration (TBC) and the Weighted Cumulative Exposure (WCE) models (PRODIGE 9 reanalysis; *n*=382)

	TG + TBC [4]	TG + WCE [5]
	Model 5 HR [95%-CI]	Model 6 (120 days) HR [95%-CI]
Control (ref.)	1	1
Maintenance group	**1.31 [1.03–1.66]**	1.23 [0.95–1.58]
Theorical Blood Concentration (1 mg/kg)	**0.77 [0.72–0.82]**	
Weighted cumulative exposure		Figure 2B
*AIC*	*2096.3*	*2102.0*
*Global Grambsch test*	*0.44*	*0.06*

*AIC* Akaike Information Criterion, *CI* Confidence Interval

The TBC model suggested that, among the patients in the maintenance group, increasing expected cumulative bevacizumab exposure by 1 mg/kg was associated with a 23% reduction (95% CI: 18 to 28% reduction) of the mortality hazard (Table 3). As such, for a patient with a good adherence to the study protocol this model implies a relative reduction of the estimated risk of death between, respectively, 88% (95%-CI=80–93%) and 97% (95%-CI=92–99%), when compared to patients who have not received any doses during the past 4 months. Accounting for a potential non-linear effect of the TBC did not improve the model fit’s according to the AIC.

#### Weighted cumulative exposure (WCE)

**Fig. 2:**
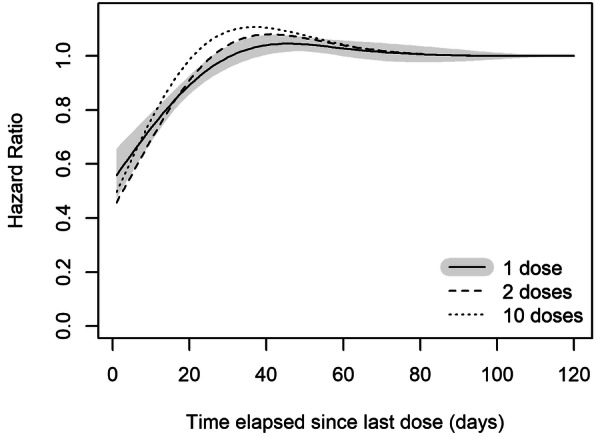
Hazard-ratios of the Weighted Cumulative Exposure (WCE) model. Changes in HRs of death with increasing time from the day of dose intake, for patients who were administered 1, 2 or 10 dose with patients who received no doses as the reference (gray shaded area indicates the 95% pointwise confidence band interval for the HR for the “1 dose” exposure)

Results for time-weighted exposure to bevacizumab are summarized in Table 3 (models 5–6). WCE model that restricted the exposure window to the past 120 days (4 months) yielded much better fit to data (AIC=2102.0) than the models with either one- or two-year window (AIC of 2121.3 and 2133.1, respectively). The estimated 120-day HRs, shown in Fig. 2, indicated a short-term protective cumulative effect of bevacizumab doses received in the last 2 to 3 weeks. In contrast, doses received more than 3 weeks ago have very little impact on the current mortality hazard, as reflected by estimated weights very close to 0 (Fig. 2). Whereas the AIC of the flexible WCE model was slightly worse than AIC of the TBC model, the moderate difference of about 5 points (Table 3) was mostly due to the higher number of degrees of freedom used to fit the WCE model. In fact, the deviances of the two models were about the same (2060.4 for TBC vs. 2062.0 for WCE) indicating that the WCE model was successful in ‘re-constructing’ the biologically plausible way effects of past bevacizumab doses cumulate over time. The estimated weight function implied that, for example, a patient with good adherence to the study protocol, had 31% lower mortality hazard than another patient in the same maintenance group who had no bevacizumab in the past 4 months.

### Sensitivity analysis

To assess the robustness of our findings, all the models were also re-estimated without adjusting for the randomization group. In this sensitivity analysis, patients of the maintenance group who did not received any bevacizumab during CFI were a priori assumed to have the same hazard as patients randomized to the control group. These analyses did not lead to important changes in the estimated effects for the different time-varying exposure metrics to bevacizumab (data not shown). When multivariable models were restricted to adjustment for only time-invariant confounders, including baseline values of truly time-varying covariates, we did not observe material changes in the estimated effects either for the TBC (HR=0.76, 95%-CI=0.70–0.81 vs HR=0.77, 95%-CI=0.72–0.82 with adjustment for updated values of time-varying covariates) or for the WCE (Appendix 8) models. Finally, when additionally adjusted for the binary time-varying indicator of “having received a dose in the last 20 days”, the best-fitting models provided similar estimates to the original analyses, without this adjustment, both for the TBC (HR=0.80;95% CI [0.70; 0.91]) or the WCE models (Appendix 8).

## Discussion

In these re-analyses of the data collected in the maintenance trial PRODIGE9 [6], we compared alternative multivariable models with different time-varying exposures to the study drug, bevacizumab. Our focus was on exploring the potential benefits of accounting for cumulative effects of past doses and their timing, in order to better understand how the past use of bevacizumab may impact survival in metastatic colorectal cancer. When we used the conventional ITT analyses, with time-invariant exposure based on randomization groups, we found no evidence of systematic differences in survival between the trial arms. In contrast, our time-varying models revealed consistently statistically significant associations between higher cumulative exposure to recent bevacizumab doses and lower risk of all-cause mortality, regardless of whether restricted only to the CFI periods or not. This pattern of results, suggests that the effects are similar whether recent bevacizumab doses are received in combination with chemotherapy or alone.

Some limitations of our analyses have to be recognized. Cumulative exposure analyses allowed us to account for the large intra group heterogeneity between the individual patterns of bevacizumab use in the maintenance group. However, as we relied on doses actually received by individualized patients, the benefits of randomization did not apply to our results. Specifically, the estimated associations between cumulative bevacizumab exposures and survival could be biased by confounding. We took into account all available potential confounding factors that could be associated with both treatment intensity/dose and the risk of death. Particularly, as the current clinical state of the patient is a strong predictor of both (i) his/her probability of receiving the treatment and (ii) the risk of death, we adjusted for all available time-varying markers of the patient’s current clinical condition. Arguably, this might have led to a different source of bias: if some of the time-varying covariates could mediate the effects of earlier bevacizumab exposure, adjusting for such mediators would bias the estimated effect of a time-varying treatment [32]. However, in sensitivity analyses, we did not observe major differences between treatment effects estimated through simplified models with only time-invariant covariates, versus our main models that adjusted for time-dependent covariates. On the other hand, our dataset did not contain information on some known risk factors*,* such as mutation status [33] or more accurate measurements of patients’ general health than the global WHO assessment, so we cannot exclude some degree of residual unmeasured confounding.

Clearly, the TBC and WCE models fit our data much better than alternative models with simpler time-varying exposure metrics. In the absence of bias, this would indicate that the effect of past bevacizumab doses varies considerably depending on how long ago they were taken [15]. Indeed, the results of both models were consistent in that the protective effect is largely driven by bevacizumab doses taken in the past 2 to 3 weeks. However, it is almost certain that these results are partly biased as the estimated protective effects are exceedingly strong (particularly for the TBC model). As such, the estimated effects probably reflect a mixture of (i) the true protective effect of the recent bevacizumab doses and (ii) lower short-term risk of death among patients who have been able to receive the treatment recently. In our analysis, the use of time-dependent variables (to adjust on the clinical state), or of a binary variable (“having received a treatment dose in the last 20 days”) were insufficient to completely disentangle between the direct effect of the treatment from the effects of patient’s current clinical characteristics associated with higher probability of receiving the treatment.

Due to its flexibility, the WCE model probably generated more reliable estimates than the TBC model, with an estimated HR comprised between 0.50 and 0.86 for patients receiving bevacizumab every 2 weeks, relative to patients who did not received bevacizumab since at least 4 months. These estimates seem generally comparable to the results of randomized control trial’s evaluating the impact of bevacizumab on overall survival. Indeed, a recent meta-analysis of relevant trials estimated the effect of the addition of bevacizumab in a first-line treatment for mCRC patients on overall survival at 16% relative risk reduction (HR = 0.84; 95% CI: 0.77–0.92), relative to first-line treatment without bevacizumab [34]. It should be noted that this estimate is not strictly comparable to ours, as the meta-analysis estimate is based on treatment groups comparisons whereas our analysis relies on a theoretical individual trajectory of exposure to treatment.

The advantages and limitations of each time-varing exposure metric used in our analysis are outlined in Appendix 9. Each metric may be of interest in particular studies, depending on the objectives, exposure, prior knowledge and/or the structure of the available data. As different metrics capture different aspects of the exposure, and complement each other, it may be preferable to interpret their results jointly, to gain a more comprehensive assessment of the relationship between exposure and outcome.

## Conclusions

In conclusion, carrying secondary analyses of RCTs using different cumulative time-varying exposure metrics to account for actual use of drugs during the trial, may be useful to complement the conventional intent-to-treat analyses. However, they cannot substitute intent-to-treat analysis, as time-varying analyses do not benefit from the randomization; their results must be interpreted with caution, all the more if the exposure is conditioned by the risk of occurrence of the event.

## Supplementary Information

**Additional file 1: Appendix 1.** Additional information about the method used for testing linearity and proportional hazards hypotheses. **Appendix 2.** Directed Acyclic Graph. **Appendix 3.** Flow-chart. **Appendix 4.** Comparison of included and excluded patients. **Appendix 5.** Description of standardized exposure updated at 60,120,182, 365 and 730 days after baseline. **Appendix 6.** Results from flexible models. **Appendix 7.** Theoretical model for bevacizumab blood concentration and observed exposure. **Appendix 8.** Weight functions for WCE models. **Appendix 9.** Main advantages and limits of exposure metrics used in this reanalysis for estimating drug effect.

## Data Availability

The datasets used and/or analysed during the current study are available from the corresponding author on reasonable request.

## Abbreviations

AIC: Akaike Information Criterion
CE: Cumulative Exposure
CFI: Chemotherapy Free Interval
CT: Computed Tomography
HR: Hazard Ratio
ITT: Intent-To-Treat
mCRC: Metastatic Colorectal Cancer
PH: Proportional Hazards
PS: Performance Status
RCT: Randomized Control Trial
TBC: Theoretical Blood Concentration
VEGF: Vascular Endothelial Growth Factor
WCE: Weighted Cumulative exposute
WHO: World Health Organization
