# Supplementary material for: Impact of time-varying cumulative bevacizumab exposures on survival: re-analysis of data from randomized clinical trial in patients with metastatic colo-rectal cancer
Source: BMC Med Res Methodol. 2021 Jan 9;21:14. doi: 10.1186/s12874-020-01202-9 (PMC7796644; doi:10.1186/s12874-020-01202-9)
Supplement: Supplementary file 1 — Additional file 1: Appendix 1. Additional information about the method used for testing linearity and proportional hazards hypotheses. Appendix 2. Directed Acyclic Graph. Appendix 3. Flow-chart. Appendix 4. Comparison of included and excluded patients. Appendix 5. Description of standardized exposure updated at 60,120,182, 365 and 730 days after baseline. Appendix 6. Results from flexible models. Appendix 7. Theoretical model for bevacizumab blood concentration and observed exposure. Appendix 8. Weight functions for WCE models. Appendix 9. Main advantages and limits of exposure metrics used in this reanalysis for estimating drug effect. [file 12874_2020_1202_MOESM1_ESM.docx]

# Figures, tables and additional files

## Appendix 1: Additional information about the method used for testing linearity and proportional hazards hypotheses

By default in the Cox proportional-hazards model, a linear relationship is assumed between a continuous covariate, including time-varying exposures, and the logarithm of the hazard (1,2). However, this hypothesis is not always true (2,3): a one-point exposure increase can have a varying effect according to the level of the exposure (low doses, high doses). In such cases the estimation produced when a linear relationship is assumed will under/overestimate the real exposure effect. Thus, we estimated the potentially non-linear effect of time-varying cumulative exposures using either quadratic B-spline with one knot or, in the case of convergence problems, a simpler, conventional quadratic function. The proportional hazards risk is the second main hypothesis of the Cox model: in its classic formulation the Cox model hypothesizes that the risk associated with an exposure is constant over time. As for the nonlinear relationship this hypothesis is not always true (for example, some tumor cells can become resistant to targeted therapies when mutations occur which will lead to a decreased effect over time) and will lead to under/overestimate the real exposure effect according to the part of the follow-up considered. To assess deviation from the proportional hazards (PH) hypothesis we used the Grambsch-Therneau test (4). If the PH hypothesis was rejected, we fitted models with time-varying coefficients using either a quadratic B-spline with one knot (2) or, if the spline model did not converge, a piecewise Cox model (5), with follow-up split into two periods (<6 vs. > 6 months after time 0). The flexible extensions of the Cox model make it possible to take into account non-linearity and non-proportional risk jointly, thus better represent the relationship between the exposure and the outcomes in case of nonlinear/non proportional effects. They generally lead to a better data fit and do not need to make hypothesis to account for these deviations (contrary to other approaches, such as categorizing a continuous exposure or splitting the follow-up in time period). The main limitations of flexible models are : a large sample size/number of events is needed to their estimation, they can lead to overfit, their calculation can be time-consuming and the interpretation of non-linear and non-proportional effects can be challenging.

References:

1. Cox DR. Regression Models and Life-Tables. J R Stat Soc Ser B Methodol. 1972;34(2):187–220.

2. Abrahamowicz M, MacKenzie TA. Joint estimation of time-dependent and non-linear effects of continuous covariates on survival. Stat Med. 2007 Jan 30;26(2):392–408.

3. Hastie T, Tibshirani R. Varying-coeffcient models (with discussion). J R Stat Soc B. 1993;(55):757–96.

4. Grambsch PM, Therneau TM. Proportional Hazards Tests and Diagnostics Based on Weighted Residuals. Biometrika. 1994;81(3):515–26.

5. Moreau T, O’Quigley J, Mesbah M. A Global Goodness-of-Fit Statistic for the Proportional Hazards Model. J R Stat Soc Ser C Appl Stat. 1985;34(3):212–8.

## Appendix 2: Directed Acyclic Graph

Sex/Age at diagnosis

Treatment administration

Clinical state

Survival (outcome)

Progression

Tumour location

WHO performance status, weight, anemia…

Toxicities

Hospitalization

Unaccounted for

Accounted for

Time invariant

Time varying parameter

Figure: Directed Acyclic Graph presenting the main risk factors and disease history for colorectal metastatic cancer patient in the PRODIGE 9 study

## Appendix 3: Flow-chart


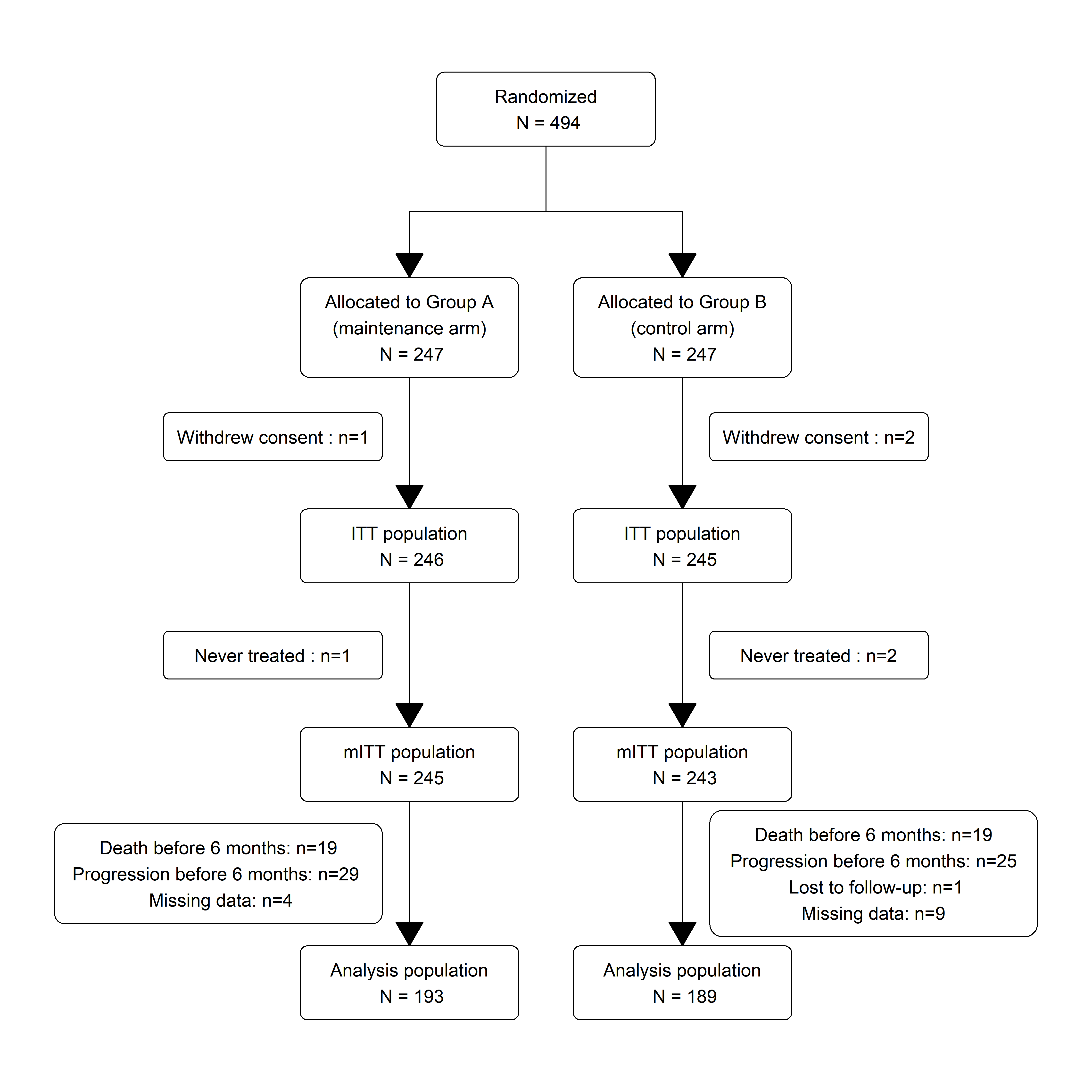


mITT : modified Intention To Treat

## **Appendix 4: Comparison of included and excluded patients**

Table: Baseline characteristics of patients included and excluded in the re-analyses of the PRODIGE 9 Trial

|  | Included patients | Excluded patients |
| --- | --- | --- |
| **Sample size** | 382 | 106 |
| **Patients characteristics** |  |  |
| Age |  |  |
| < 65 years old | 199 (52.1%) | 50 (47.2%) |
| ≥ 65 & <75 years old | 117 (30.6%) | 39 (36.8%) |
| ≥ 75 years old | 66 (17.3%) | 17 (16.0%) |
| Women | 133 (34.8%) | 41 (38.7%) |
| Baseline WHO performance status (PS) |  |  |
| 0 | 207 (54.2%) | 42 (39.6%) |
| 1 | 159 (41.6%) | 47 (44.3%) |
| 2 | 16 (4.2%) | 17 (16.0%) |
| Alkaline phosphatase > 300 U/L | 68 (17.8%) | 25 (23.6%) |
| Leucocytes > 10x10^9^ | 96 (25.1%) | 30 (28.3%) |
| Localisation* |  |  |
| Left colon | 125 (32.7%) | 28 (31.7%) |
| Right and transverse colon | 87 (22.8%) | 18 (19.4%) |
| Unspecified colon | 89 (23.3%) | 26 (28.0%) |
| Rectum | 81 (21.2%) | 21 (22.6%) |

**13 patients with missing data in the excluded group for this variable*

## **Appendix 5: Description of standardized exposure updated at 60,120,182, 365 and 730 days after baseline**


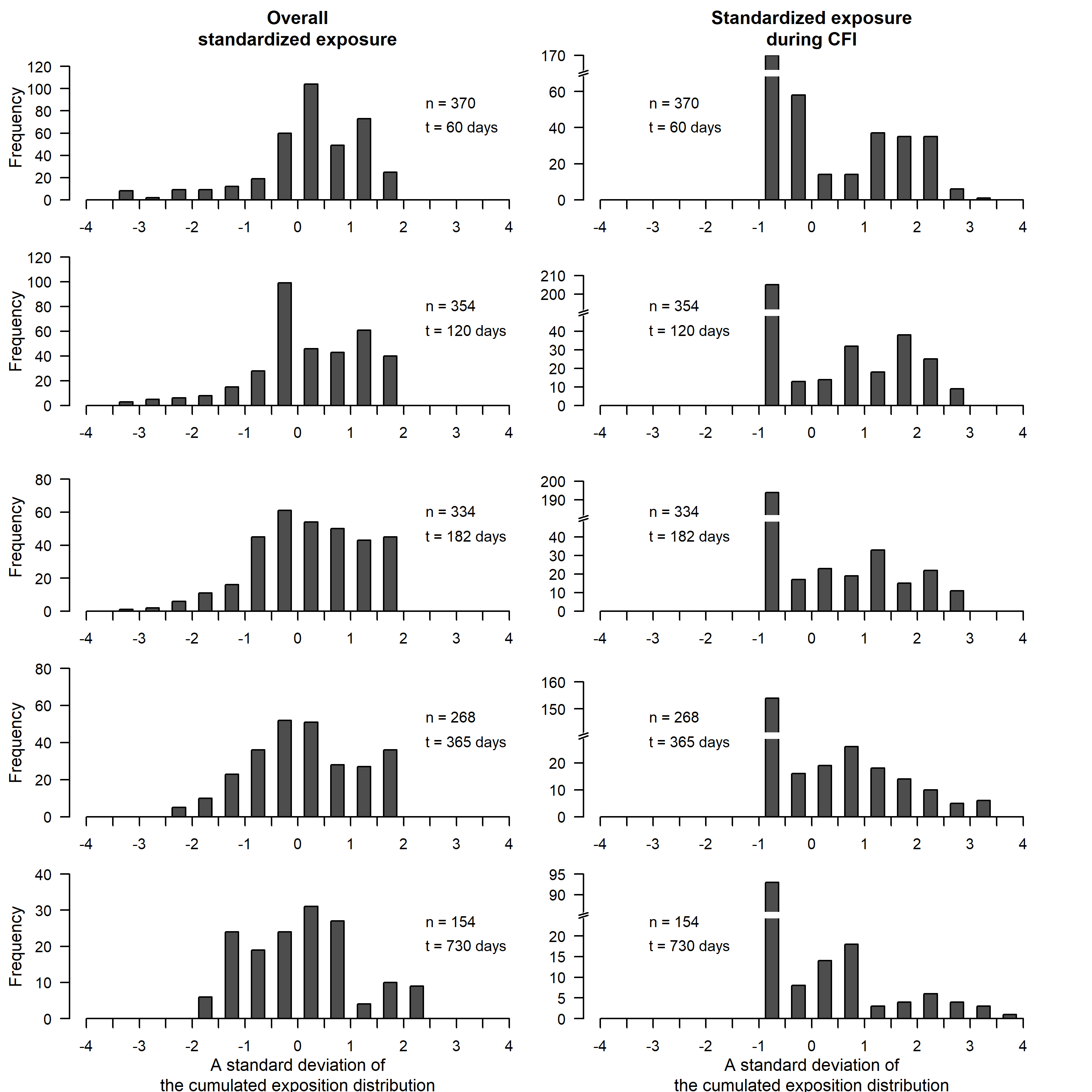


*One unit on the x-axis is equivalent to one standard deviation of the distribution of the cumulative exposure at a given time point.*

## Appendix 6: Results from flexible models


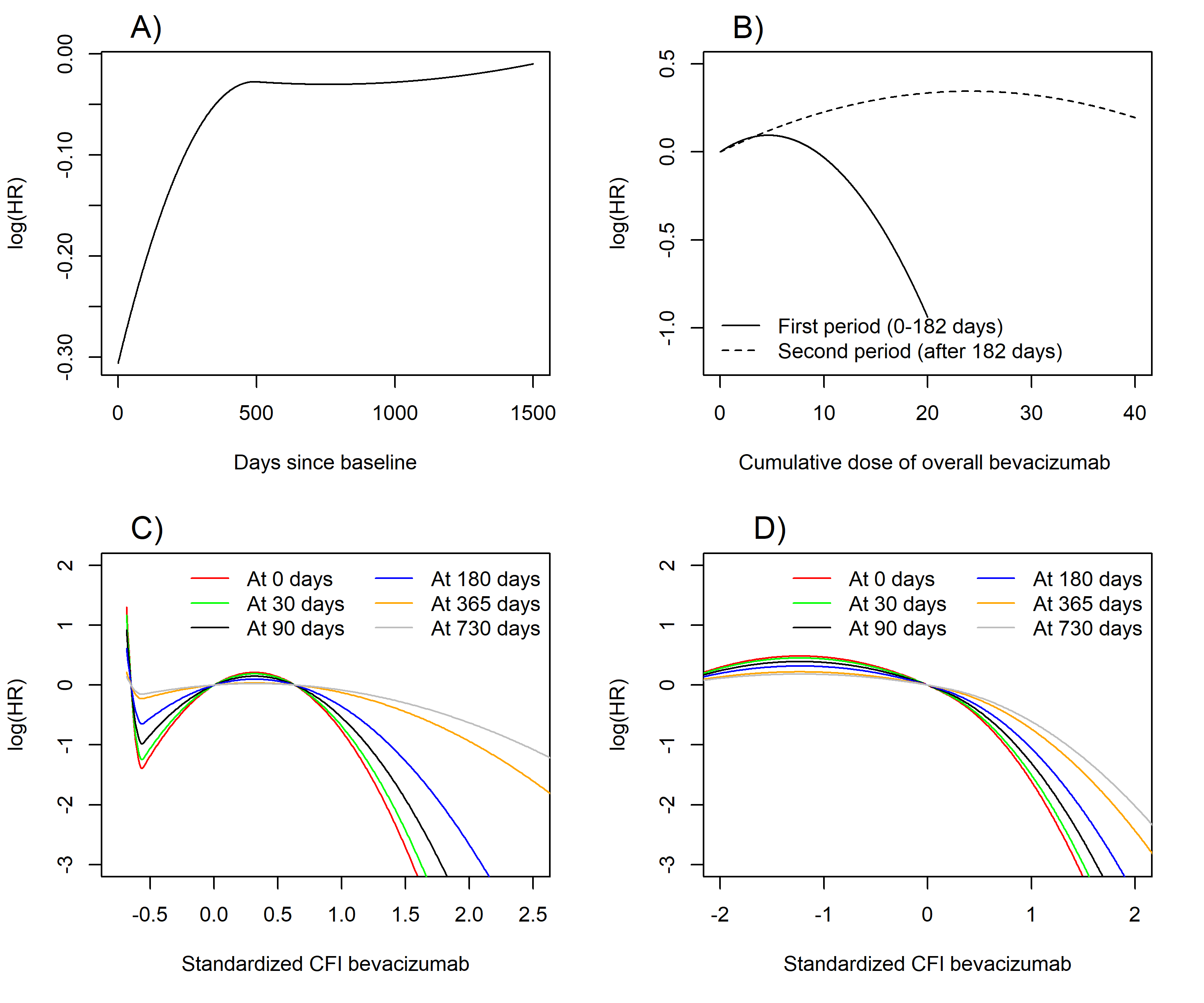


Title: Time-varying and non-linear effects of bevacizumab cumulative exposure:

1. Time-varying of the log-linear effect of the cumulative exposure to bevacizumab during Chemotherapy Free Interval –CFI (p=0.03 for the deviation from the proportional hazard hypothesis -DPH). Each additional unit increase in the cumulative dose of bevacizumab (defined as the dose received during one cure, i.e; 5 mg/kg) was associated with a reduced risk of death only during the first months after baseline (delayed in this sensitivity analysis 6 month after randomization).
2. Time-varying and non-loglinear effect of the overall cumulative exposure to bevacizumab (AIC=2203.3; p=0.02 for DPH). We restricted the maximum dose to 20 points in the first period as most patients did not attain this dose in this period. The protective effect associated with each additional unit increase in cumulative bevacizumab dose was essentially observed in the first period (first 6 months following baseline).
3. Time-varying and non-loglinear effect of the standardized cumulative exposure to bevacizumab during CFI (AIC=2183.4 ; p=0.01 for DPH). The non-loglinear effect (*modeled by a quadratic function)* was mostly characterized by a higher risk of death for low standardized doses (patients of the maintenance group who did not receive any dose) followed by a low to moderate risk of death for low to moderate doses and by a subsequent decreased risk for the highest cumulative standardized dose (similar to what was observed in A). However, this effect was reduced after 6 months from baseline.
4. Time-varying and non-linear effect of the standardized overall cumulative exposure to bevacizumab (AIC=2180.8; p=0.01 for DPH). The non-loglinear (modeled by a quadratic B-Spline with one knot*)* and non-proportional effect of the overall standardized cumulative exposure to bevacizumab followed the same pattern than that observed in B.

## Appendix 7: Theoretical model for bevacizumab blood concentration and observed exposure


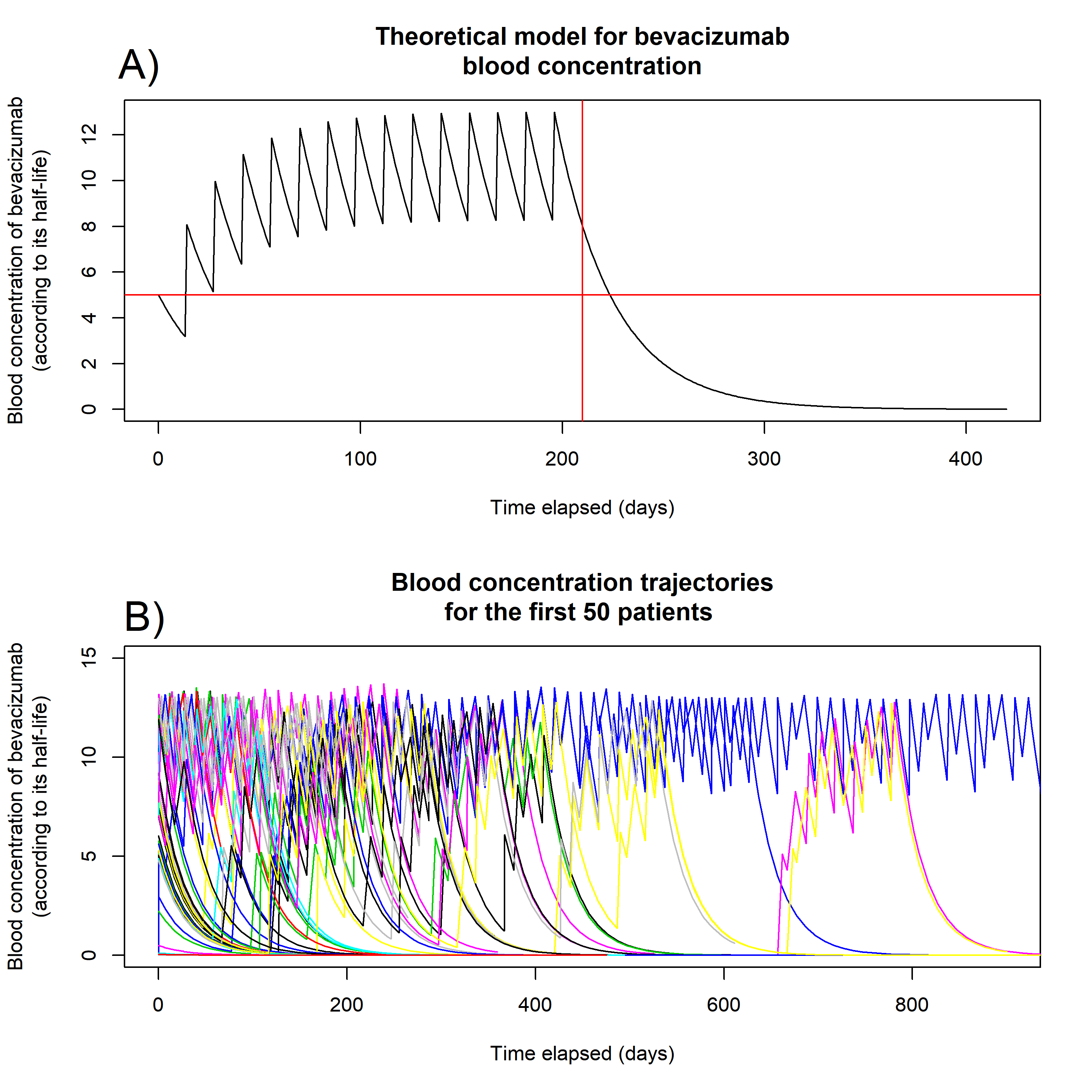
Figure: Theoretical concentration variation over time for A) a fictitious patient undergoing 15 cures every two weeks and no further dose B) for the first fifty patients included in the PRODIGE 9 study.

The figure and table are presented in this annex to illustrate the variation of the theoretical blood concentration over time. A fictitious patient is used to illustrate the initial increase in blood concentration and the subsequent stabilization after approximatively 3-4 cures (figure A and table). In figure B we showed the variations of blood concentration for the first fifty patients included to illustrate the variability observed in the study : some patients maintained a high concentration throughout the whole 3-year follow-up (patients of the maintenance group with high observance and no drop-out), some patients experienced period with no blood concentration followed by period with high concentration (patients of the control group who had a progression during a CFI) and for a significant part of patients we observed a decrease in blood concentration immediately after the induction sequence and no subsequent increase (drop-out of the study during the first cure-free interval)

Table: Example of the calculation of the Theorical Blood Concentration of bevacizumab for a specific individual according to the exponential decay model [4] with a half-life h=20

|  |  |  |  |  |  |  |  |  |  |  |  |  |  |  |  |  |  |  |  |
| --- | --- | --- | --- | --- | --- | --- | --- | --- | --- | --- | --- | --- | --- | --- | --- | --- | --- | --- | --- |
| Bevacizumab | D0 | D7 | D14 | D21 | D28 | D35 | D42 | D49 | D56 | D63 | D70 | D77 | D84 | D91 | D98 | D105 | D112 | D119 | D126 |
| 1st cure | 5.000 | 3.923 | 3.078 | 2.415 | 1.895 | 1.487 | 1.166 | 0.915 | 0.718 | 0.563 | 0.442 | 0.347 | 0.272 | 0.213 | 0.167 | 0.131 | 0.103 | 0.081 | 0.063 |
| 2nd cure |  |  | 5.000 | 3.923 | 3.078 | 2.415 | 1.895 | 1.487 | 1.166 | 0.915 | 0.718 | 0.563 | 0.442 | 0.347 | 0.272 | 0.213 | 0.167 | 0.131 | 0.103 |
| 3rd cure |  |  |  |  | 5.000 | 3.923 | 3.078 | 2.415 | 1.895 | 1.487 | 1.166 | 0.915 | 0.718 | 0.563 | 0.442 | 0.347 | 0.272 | 0.213 | 0.167 |
| 4th cure |  |  |  |  |  |  | 5.000 | 3.923 | 3.078 | 2.415 | 1.895 | 1.487 | 1.166 | 0.915 | 0.718 | 0.563 | 0.442 | 0.347 | 0.272 |
| 5th cure |  |  |  |  |  |  |  |  | 5.000 | 3.923 | 3.078 | 2.415 | 1.895 | 1.487 | 1.166 | 0.915 | 0.718 | 0.563 | 0.442 |
| 6th cure |  |  |  |  |  |  |  |  |  |  | 5.000 | 3.923 | 3.078 | 2.415 | 1.895 | 1.487 | 1.166 | 0.915 | 0.718 |
| 7th cure |  |  |  |  |  |  |  |  |  |  |  |  | 5.000 | 3.923 | 3.078 | 2.415 | 1.895 | 1.487 | 1.166 |
| 8th cure |  |  |  |  |  |  |  |  |  |  |  |  |  |  | 5.000 | 3.923 | 3.078 | 2.415 | 1.895 |
| 9th cure |  |  |  |  |  |  |  |  |  |  |  |  |  |  |  |  | 5.000 | 3.923 | 3.078 |
| 10th cure |  |  |  |  |  |  |  |  |  |  |  |  |  |  |  |  |  |  | 5.000 |
| Theorical Blood Concentration (mg/kg) | 5.000 | 3.923 | 8.078 | 6.338 | 9.973 | 7.824 | 11.139 | 8.739 | 11.857 | 9.303 | 12.299 | 9.649 | 12.571 | 9.863 | 12.738 | 9.994 | 12.841 | 10.075 | 12.905 |

## Appendix 8: Weight functions for WCE models


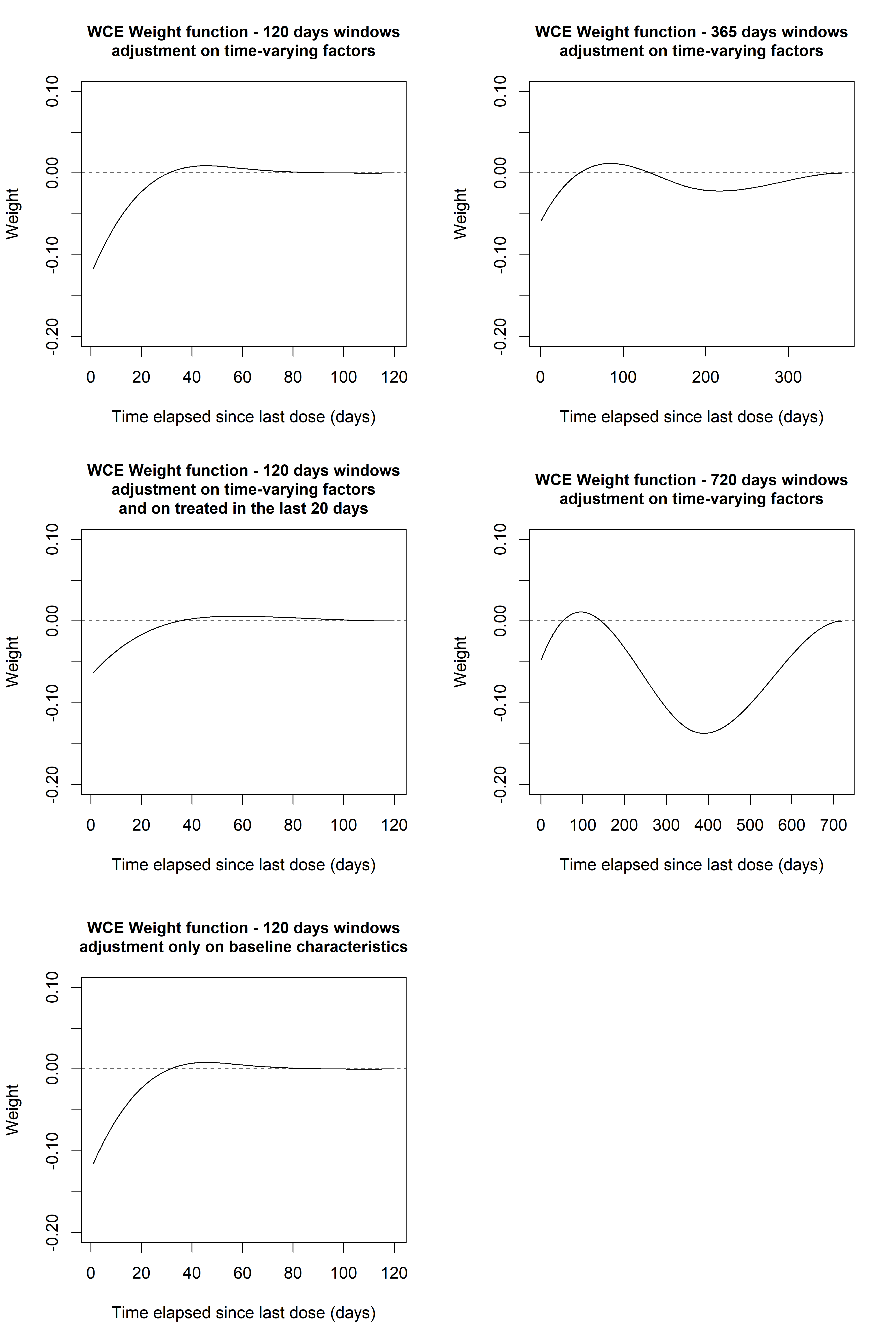


## Appendix 9: Main advantages and limits of exposure metrics used in this reanalysis for estimating drug effect

| **Exposure Metric** | **Advantages** | **Limitations** |
| --- | --- | --- |
| **Cumulative continuous exposure (CE)** | - Simple calculations  - Easy interpretation  - May be efficient (1 degree of freedom) for testing the hypothesis of no association | - Range of exposure varies over time: a one-point difference between two patients will probably not produce the same effect at the beginning or at the end of the follow-up, leading to a non-proportional and non-loglinear effect, requiring complex modeling that in our example failed to control bias - Does not account for the delay since administration |
| **CE categorized by quantiles (CEQ)** | - Easier interpretation of non-log-linear relationship compared to CE (data range stable through time)  - Allow to differentiate specifically individual who did not receive any dose, from patients with limited drug exposure; both being with characteristics requiring specific attention (contrary to StCE) - Easier to communicate than StCE | - Requires defining time-varying thresholds - Does not account for the delay since administration |
| **Standardized CE (StCE)** | - Easier interpretation of non-log-linear relationship compared to CE (data range stable through time) - Often less parameters needed than CEQ and does not need to define threshold | - Requires a transformation and to use standard deviation of a time-varying distribution as a unit (may be difficult to communicate) - Does not account for the delay since administration |
| **Theoretical Blood Concentration (TBC)** | - Accounts for delays since drug administration - May account for differences in blood concentration estimates according to patients characteristics (not used in our analysis)  - If available, reflects well established pharmacodynamics of the drug - Simpler to calculate than WCE  - Less parameters (df’s) needed than WCE | - Requires knowledge about the dynamics describing the decrease over time in blood concentration of the drug - TBC estimates may not reflect the actual target concentrations (blood, tissue...) and can mis-represent the true effect of the drug if this effect is (partly or entirely) lagged rather than acute, or if elimination in study subjects is e.g. slower (due to advanced age) than expected |
| **Weighted Cumulative Exposure (WCE)** | - Accounts for delays since drug administration - R package is available - Most flexible of all the metrics  - No prior knowledge needed to estimate the weight function  - Reflects directly the associations observed in the empirical data  - Validated in extensive simulations | - Requires testing different models (time windows, spline parameters) - Can lead to overfitting bias, especially in smaller samples - Need many events to produce stable estimates - Longer computational time and large memory necessary for large datasets |
